# Prostate cancer-derived urine exosomes: a novel approach to biomarkers for prostate cancer

**DOI:** 10.1038/sj.bjc.6605058

**Published:** 2009-04-28

**Authors:** J Nilsson, J Skog, A Nordstrand, V Baranov, L Mincheva-Nilsson, X O Breakefield, A Widmark

**Affiliations:** 1 Department of Radiation Sciences, Oncology, Umeå University, Umea, Sweden; 2 Neuro-oncology Research Group, Cancer Center Amsterdam, Department of Neurosurgery, VU Medical Center, Amsterdam, The Netherlands; 3 Departments of Neurology and Radiology, Massachusetts General Hospital, and Neuroscience Program, Harvard Medical School, Boston, MA, USA; 4 Department of Clinical Immunology, Umeå University, Umea, Sweden

**Keywords:** prostate cancer, prostasomes, tumour exosomes, biomarkers

## Abstract

Herein, we describe a novel approach in the search for prostate cancer biomarkers, which relies on the transcriptome within tumour exosomes. As a proof-of-concept, we show the presence of two known prostate cancer biomarkers, *PCA-3* and *TMPRSS2:ERG* the in exosomes isolated from urine of patients, showing the potential for diagnosis and monitoring cancer patients status.

Prostate cancer (PCa) is the only one of the four solid tumour types, breast, lung and colorectal cancer, which has a clinical useful protein biomarker for diagnostics and follow-up after treatment. Prostate-specific antigen (PSA) has shown reasonable sensitivity for detection of incipient cancer and can also predict response to treatment (Freedland *et al*, 2005). One of the drawbacks with PSA is its low specificity, such that benign hyperplasic conditions can also be associated with a PSA increase (Catalona *et al*, 1994). Thus, additional PCa biomarkers are needed, especially the ones that give information about the severity of the disease and can predict high or low risk for future metastases.

In this article, we evaluated a novel approach to find predictive markers for PCa, and analysed RNA in urine exosomes from PCa patients. Two types of microvesicles are present in prostate secretions: (1) prostasomes (150–500 nm), produced by prostatic ductal epithelial cells that are a normal component of seminal fluid and play a role in male fertility (Burden *et al*, 2006); and (2) exosomes, specialised nanovesicles (30–100 nm) with a cup-shaped morphology, actively secreted by a variety of normal and tumour cells. Elevated exosome secretion has been found in malignancy effusions, serum and urine from cancer patients (Mitchell *et al*, 2009). In addition, it was shown that exosomes released from a mast cell line (Valadi *et al*, 2007) and glioblastoma tumour cells (Skog *et al*, 2008) contain intact mRNA that can be transferred to recipient cells and therein translated into functional proteins (Skog *et al*, 2008). Certain RNA transcripts are enriched several 100-fold in the exosomes compared with the donor cells, supporting a specific packing mechanism (Skog *et al*, 2008). The exosomes lack essentially all of the ribosomal RNA, which represent ∼80% of the total RNA in cells, and thus contains mainly mRNAs and microRNAs (miRNAs). Therefore, exosomes are enriched in unique transcripts specific to tumour cells that may be below detection limit even in the tumour cells themselves (Skog *et al*, 2008; Taylor and Gercel-Taylor, 2008). In addition, individual PCa are often very heterogeneous in their phenotype, and a biopsy taken from one region of the tumour can have a distinct genotype from another region. Therefore, analysing the transcriptome in secreted PCa exosomes in urine has the advantages of being both noninvasive and informative as to the overall tumour malignancy status, including tumour-specific splice variants, mutations, and mRNA and miRNA levels known to be diagnostic for PCa.

Other groups have shown that tumour cells shed into the urine after mild prostate massage can be used as an mRNA source for the discovery of tumour biomarkers (Hessels *et al*, 2007; Laxman *et al*, 2008). In this study we have taken this concept a step further to analyse the tumour exosome fraction in urine. Using a nested PCR-based approach, we were able to show that tumour exosomes carry genetic information specific for PCa, and as a proof-of-principle we used tumour exosomes to detect two PCa mRNA biomarkers, *PCA-3* and *TMPRSS2:ERG* (Hessels *et al*, 2007; Laxman *et al*, 2008). This study supports the use of RNA in exosomes isolated from urine as diagnostic markers for PCa, and offers an alternative, sensitive and unique new type of PCa biomarkers.

## Materials and methods

### Sample acquisition and microvesicle preparation

Samples were obtained after informed consent. Urine from the patients was collected before and after receiving prostate massage, except for patients with bone metastases where only a normal urine sample was collected. Detailed information about the patients is presented in Table 1. The microvesicular fraction was prepared by differential centrifugation. First, cells were pelleted at 500 *g* for 20 min at 10°C and discarded, and then additional cellular debris was removed by centrifugation at 16 000 **g** for 20 min at 10°C, followed by filtration through a 0.45 *μ*m filter device (Millipore, Bedford, MA, USA). The microvesicles in the filtrate were then pelleted by ultracentrifugation (Beckman Coulter AB, Bromma, Sweden; Ti70 rotor) at 100 000 **g** for 90 min at 10°C. For electron microscopic studies, the microvesicles were additionally purified by ultracentrifugation in a 20 and 40% sucrose gradient and washed with filtered phosphate-buffered saline (PBS).

### Electron microscopy

To maximally concentrate the microvesicles, 15 *μ*l drops in PBS were placed on 2% agarose. Formvar/carbon-coated glow-discharged nickel grids were placed on top of the drops and allowed to stand for 5–10 min to absorb excess fluid. The grids with adherent microvesicles were washed and fixed with 2% paraformaldehyde in PBS for 10 min. For negative staining, the grids were incubated on ice for 10 min with 25 *μ*l drops of 1.9% methyl cellulose containing 0.3% uranyl acetate. Excess fluid was removed before examination with electron microscope. For immunoelectron microscopy, the microvesicles were incubated for 10 min in blocking solution (0.1 M glycine and 0.3% bovine serum albumin) and added to the grids. The microvesicles were incubated with anti-CD63 monoclonal antibodies (Fitzgerald laboratories, Concord, MA, USA) or with isotype-matched murine control antibodies (Dako A/S, DakoCytomation Norden, Glostrup, Denmark) as a negative control for 1 h. After washing, the grids were incubated with goat-anti-mouse secondary antibodies conjugated with 10 nm-sized gold particles (Sigma-Aldrich Sweden AB, Stockholm, Sweden) for 1 h. After washing and additional fixation in 2.5% glutaraldehyde, the grids were negatively stained as described above.

### Total RNA isolation, cDNA synthesis and nested PCR analysis

Total RNA was extracted from the exosomal or prostasomal pellet and treated with DNase according to the manufacturer's recommendations (RNAqueous and TURBO-DNase, Ambion Inc., TX, USA), and later converted to cDNA (RETROscript, Ambion). Nested PCR was used to detect transcripts of the selected biomarkers. In brief, 1.5 *μ*l of the cDNA product was amplified in a 15 *μ*l reaction, using AmpliTaq DNA polymerase (Applied Biosystems, Foster City, CA, USA) with the F1/R1 primer set for 40 cycles, then 1.5 *μ*l from the first PCR reaction were amplified with the F2/R2 primer set for another 40 cycles. Positive samples from the nested PCR reaction were then re-amplified from the first PCR reaction for a second time and digested with restriction enzymes to ensure correct products. The *TMPRSS2:ERG* PCR products were re-amplified with the F2/R2 primers, and the product was digested with Hae-II (New England Biolabs, lpswich, MA, USA) generating two distinct bands of 54 bp and 68 bp; the *PCA-3* PCR products were re-amplified with the F1/R2 primers and digested with Sca-I (New England Biolabs), generating two fragments of 90 bp and 62 bp. Each gene product was also cloned into a cloning vector (pCR4-TOPO, Invitrogen Ltd, Paisley, UK) and sequenced (BigDye, Applied Biosystems) to further verify the correct gene product. Primers used are summarised in Table 2.

## Results

### mRNA analysis of the microvesicular fraction

The patients enroled in this study (Table 1) were divided into four groups; newly diagnosed without receiving any treatment, diagnosed and under androgen deprivation therapy (ADT) and patients with verified bone metastases or patients selected for EM evaluation. The newly diagnosed cases had not received any kind of therapeutic treatment, and had detectable *PSA* mRNA expression within the urine exosomal fraction (data not shown). In the newly diagnosed group, two out of the four urine samples were negative for *PSA* mRNA transcripts, before mild prostate massage, whereas all were positive after mild prostate massage (data not shown), indicating that mild prostate massage increased the exosomal secretion into the urethra and subsequently into the collected urine fraction.

**Figure 1:**
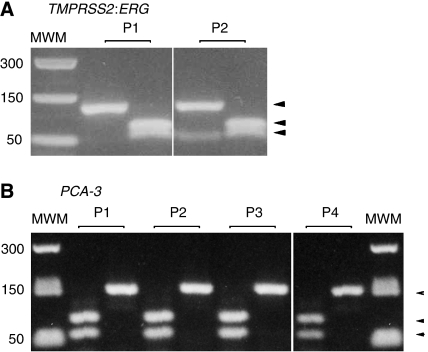
Restriction enzyme analysis of the positive PCR products. (**A**) Positive tumour samples (P1 and P2) for the *TMPRSS2:ERG* gene fusion showing fragment sizes after digestion with HaeII, 68 bp and 54 bp (right lane) and the undigested product, 122 bp (left lane). (**B**) Positive samples for the *PCA-3* gene product before and after digestion with Sca1, fragment sizes after digestion with Sca1 were 90 bp and 62 bp (left lane), and the uncut fragment size was 152 bp (right lane).

The mRNA transcripts for the fusion gene *TMPRSS2:ERG* were detected in two out of the four patients who had a high Gleason score and PSA levels, and not in the two low-risk tumours (patient 3 and 4), whereas *PCA-3* transcripts were detected in all of the patients after mild prostate massage (Table 1). This is in accordance with the published finding on PCa biopsies and from tumour cells in urine (Bussemakers *et al*, 1999; Nam *et al*, 2007; Tu *et al*, 2007; van Gils *et al*, 2007). The gene products were analysed with restriction analysis (Figure 1) together with sequencing of the products to confirm that the positive bands corresponded to the appropriate mRNAs (data not shown).

Neither of the ADT patients group (patient 5–6) or the patients with verified bone metastases (patient 7–9) had detectable *PSA* mRNA levels or were positive for *PCA-3* or *TMPRSS2:ERG* (Table 1). The loss of biomarker expression in the ADT patient group correlated with tumour regression and a positive response to the ADT. The patients with bone metastases had an impaired/nonfunctional prostate, either after medical castration (patient 8–9) or radical prostatectomy (patient 7). Taken together, these results show the potential of developing a new method of diagnosis for PCa by analysis of tumour-specific RNA in tumour exosomes in urine.

### Electron microscopy of the microvesicular fraction

**Figure 2:**
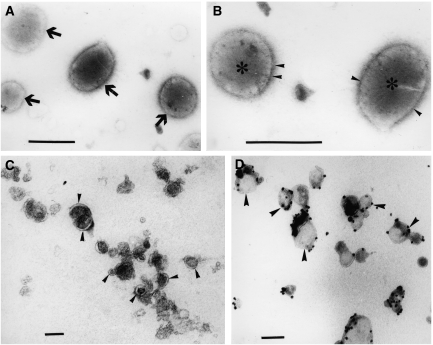
Electron microscopy of microvesicles isolated from urine of healthy donor (**A** and **B**) and PCa patient (patient nr. 11, **C** and **D**). (**A**) Microvesicles from healthy donor displaying the typical size (150–500 nm) and ultrastructure of electron-dense and electron-lucent prostasomes (arrows). (**B**) At high magnification, note the limiting membrane (arrowheads) and the unstructured matrix (star) of prostasomes. (**C**) Urine microvesicles from PCa patient showing small size (30–100 nm) and cup-shaped morphology (arrowheads) characteristic of exosomes. (**D**) Immunoelectron micrograph showing the presence of the typical surface exosomal marker CD63 on the microvesicles (arrowheads). Bars: **A**, **B** 500 nm; **C**, **D** 100 nm.

Urine microvesicles from one patient with a low-grade tumour (patient 10), one patient with a locally high-grade tumour (patient 11) and one healthy young volunteer were analysed by electron microscopy. Figures 2A and B illustrate the microvesicular urine fraction of the healthy donor. Two types of typical 500 nm-sized prostasomes are seen – ‘dark’ prostasomes with electron-dense contents and inclusions, and ‘light’, less dense ones. They were CD63 negative after immunogold staining (not shown). In contrast, microvesicles with cup-shaped morphology and size of 30–100 nm, typical for exosomes, were shown in the microvesicular urine fraction from the high-grade tumour (Figure 2C). Their exosomal nature was confirmed by immunoelectron microscopy after anti-CD63 gold staining (Figure 2D). The visual impression was that the exosome amount was enriched after prostate massage (not shown). It is interesting that, prostasomes were not found in the urine of PCa patients and vice versa; exosomes were not present in the urine of healthy donors. No exosomes or prostasomes were found in the PCa patient with the low-grade tumour (not shown). From these experiments we conclude that PCa-derived exosomes are present in the urine of PCa patients and that these can be used for analysis of the tumour transcriptome.

## Discussion

To validate the concept of urine exosomes as carriers of genetic information and a potential source of new cancer biomarkers, especially for PCa, we carried out a pilot study to investigate whether we could amplify two prognostic mRNA biomarkers. One of these has been shown to be overexpressed in PCa-*PCA-3* (de Kok *et al*, 2002) and the other is a product of a chromosomal rearrangement, which creates a common prostate cancer-specific product, the *TMPRSS2:ERG* fusion (Tomlins *et al*, 2005). By reverse transcription–PCR analysis of RNA in the exosome fraction from PCa patients, we were able to monitor the status of these two established biomarkers from a very limited amount of exosomal RNA. This study establishes the potential to use the RNA content of tumour exosomes derived from urine to provide a window into the tumour status, both with respect to tumour genotype/phenotype and metastatic potential. This delineation of the tumour genotype/phenotype can provide information to the clinician both with respect to diagnosis and treatment paradigms (Skog *et al*, 2008). Potentially the response to ADT could also be followed, assuming that regression of the tumour resulted in less/no expression of the biomarkers, but the loss of expression could also be because of the reduced expression of androgen-responsive genes, like *TMPRSS2* and *PCA3* (Tomlins *et al*, 2005; Shaw *et al*, 2007). That is an important distinction and further studies are needed to address this question.

Tumour exosomes are distinct from exosomes shed by normal cells, in particular, they are more abundant in cancer patients, and the exosomes shed from tumours seems to have an important role in the increased tumour growth, angiogenesis and the escape from the immune-surveillance (Abusamra *et al*, 2005; Liu *et al*, 2006; Skog *et al*, 2008). RNA extracted from the tumour exosomes contains a ‘snap-shot’ of the tumour transcriptome (Skog *et al*, 2008). Using exosomes from urine we can then, potentially, extend the available biomarkers to include mRNAs and miRNAs, not only for cancer detection, but also to try to classify the severity of the tumour phenotype and follow the tumour response to treatment. The results presented within this pilot study warrant further research to determine the use of urine exosomes in diagnostics and tumour surveillance of PCa.

## Figures and Tables

**Table 1:** Diagnostic T-stage, Gleason score, PSA levels (ng ml^–1^) before start of treatment

		**Diagnostic stage (before any treatment)**			**PCa biomarkers (in exosome fraction)**	
**Group**	**Patient**	**Grade**	**Gleason**	**PSA**	**TMPRSS2:ERG**	**PCA-3**
Untreated	P1	T3,Nx,M0	9	25	+	+
	P2	T2c,Nx,M0	7	24	+	+
	P3	T2,Nx,M0	6	7	−	+
	P4	T2,Nx,M0	6	4	−	+
						
ADT treated						
	P5	T3,Nx,M0	7	13	−	−
	P6	T2,Nx,M0	7	3	−	−
						
Verified bone metastases						
	P7^#^	T2,Nx,M0	7	11	−	−
	P8^*^	T3,Nx,M1	8	64	−	−
	P9^*^	T2,Nx,M0	8	NA	−	−
						
EM						
	P10	T1c,Nx,M0	7	14	NA	NA
	P11	T3,Nx,M0	8	5	NA	NA

ADT=androgen deprivation therapy; NA=not analysed/not available; PCa=prostate cancer; PSA=prostate-specific antigen.

Patients grouped into four categories; untreated, treated with androgen deprivation therapy/medical castration (ADT), patients with verified bone metastases, either medically castrated^*^ or prostatectomised^#^, and patients for electron microscopy (EM). Detected PCa biomarkers among the patients enroled in pilot study.

**Table 2:** Sequences of primers used for the different PCR applications and their Genbank accession number

**Gene name**	**Gene name abbreviation**	**Genbank accession number**	**Sequence**	**Primer source**
TMPRSS2/ERGa fusion transcript	*TMPRSS2:ERG*	DQ204772	F1 5′-TAGGCGCGAGCTAAGCAGGAG-3′	(Tomlins *et al*, 2005; Tu *et al*, 2007)
			F2 5′-CGCGAGCTAAGCAGGAGGC-3′	(Tu *et al*, 2007)
			R1 5′-GGGGGTTGAGACAGCCAATC-3′	(Rozen and Skaletsky, 2000)
			R2 5′-GTAGGCACACTCAAACAACGACTGG-3′	(Tomlins *et al*, 2005; Tu *et al*, 2007)
Prostate cancer antigen 3	*PCA-3*	AF103907	F1 5′- AGTCCGCTGTGAGTCT-3′	(Rozen and Skaletsky, 2000)
			F2 5′-ATCGACGGCACTTTCTGAGT-3′	
			R1 5′-CCATTTCAGCAGATGTGTGG-3′	
			R2 5′-TGTGTGGCCTCAGATGGTAA-3′	

The primers were generated using PRIMER-3 (Rozen and Skaletsky, 2000), or found in the literature as described under primer source. F2 and R2 refer to the nested primers.

## References

[bib1] Abusamra AJ, Zhong Z, Zheng X, Li M, Ichim TE, Chin JL, Min WP (2005) Tumor exosomes expressing Fas ligand mediate CD8+ T-cell apoptosis. Blood Cells Mol Dis 35: 169–173 16081306 10.1016/j.bcmd.2005.07.001

[bib2] Burden HP, Holmes CH, Persad R, Whittington K (2006) Prostasomes—their effects on human male reproduction and fertility. Hum Reprod Update 12: 283–292 16373403 10.1093/humupd/dmi052

[bib3] Bussemakers MJG, van Bokhoven A, Verhaegh GW, Smit FP, Karthaus HFM, Schalken JA, Debruyne FMJ, Ru N, Isaacs WB (1999) DD3:a new prostate-specific gene, highly overexpressed in prostate cancer. Cancer Res 59: 5975–5979 10606244

[bib4] Catalona WJ, Hudson MA, Scardino PT, Richie JP, Ahmann FR, Flanigan RC, deKernion JB, Ratliff TL, Kavoussi LR, Dalkin BL, Waters WB, MacFarlane MT, Southwick PC (1994) Selection of optimal prostate specific antigen cutoffs for early detection of prostate cancer: receiver operating characteristic curves. J Urol 152: 2037–2042 7525995 10.1016/s0022-5347(17)32300-5

[bib5] de Kok JB, Verhaegh GW, Roelofs RW, Hessels D, Kiemeney LA, Aalders TW, Swinkels DW, Schalken JA (2002) DD3PCA3, a very sensitive and specific marker to detect prostate tumors. Cancer Res 62: 2695–2698 11980670

[bib6] Freedland SJ, Mangold LA, Walsh PC, Partin AW (2005) The prostatic specific antigen era is alive and well: prostatic specific antigen and biochemical progression following radical prostatectomy. J Urol 174: 1276–1281 16145392 10.1097/01.ju.0000173907.84852.ec

[bib7] Hessels D, Smit FP, Verhaegh GW, Witjes JA, Cornel EB, Schalken JA (2007) Detection of TMPRSS2-ERG fusion transcripts and prostate cancer antigen 3 in urinary sediments may improve diagnosis of prostate cancer. Clin Cancer Res 13: 5103–5108 17785564 10.1158/1078-0432.CCR-07-0700

[bib8] Laxman B, Morris DS, Yu J, Siddiqui J, Cao J, Mehra R, Lonigro RJ, Tsodikov A, Wei JT, Tomlins SA, Chinnaiyan AM (2008) A first-generation multiplex biomarker analysis of urine for the early detection of prostate cancer. Cancer Res 68: 645–649 18245462 10.1158/0008-5472.CAN-07-3224 PMC2998181

[bib9] Liu C, Yu S, Zinn K, Wang J, Zhang L, Jia Y, Kappes JC, Barnes S, Kimberly RP, Grizzle WE, Zhang HG (2006) Murine mammary carcinoma exosomes promote tumor growth by suppression of NK cell function. J Immunol 176: 1375–1385 16424164 10.4049/jimmunol.176.3.1375

[bib10] Mitchell PJ, Welton J, Staffurth J, Court J, Mason MD, Tabi Z, Clayton A (2009) Can urinary exosomes act as treatment response markers in prostate cancer? J Transl Med 7: 4 19138409 10.1186/1479-5876-7-4 PMC2631476

[bib11] Nam RK, Sugar L, Yang W, Srivastava S, Klotz LH, Yang LY, Stanimirovic A, Encioiu E, Neill M, Loblaw DA, Trachtenberg J, Narod SA, Seth A (2007) Expression of the TMPRSS2:ERG fusion gene predicts cancer recurrence after surgery for localised prostate cancer. Br J Cancer 97: 1690–1695 17971772 10.1038/sj.bjc.6604054 PMC2360284

[bib12] Rozen S, Skaletsky H (2000) Primer3 on the WWW for general users and for biologist programmers. Methods Mol Biol 132: 365–386 10547847 10.1385/1-59259-192-2:365

[bib13] Shaw G, Oliver RTD, Purkiss T, Prowse DM (2007) Re: Christine McKillop. Interview with Jack Schalken: PCA3 and its use as a diagnostic test in prostate cancer. Eur Urol 51: 860–862 17049717 10.1016/j.eururo.2006.08.053

[bib14] Skog J, Wurdinger T, van Rijn S, Meijer DH, Gainche L, Sena-Esteves M, Curry Jr WT., Carter BS, Krichevsky AM, Breakefield XO (2008) Glioblastoma microvesicles transport RNA and proteins that promote tumour growth and provide diagnostic biomarkers. Nat Cell Biol 10: 1470–1476 19011622 10.1038/ncb1800 PMC3423894

[bib15] Taylor DD, Gercel-Taylor C (2008) MicroRNA signatures of tumor-derived exosomes as diagnostic biomarkers of ovarian cancer. Gynecol Oncol 110: 13–21 18589210 10.1016/j.ygyno.2008.04.033

[bib16] Tomlins SA, Rhodes DR, Perner S, Dhanasekaran SM, Mehra R, Sun XW, Varambally S, Cao X, Tchinda J, Kuefer R, Lee C, Montie JE, Shah RB, Pienta KJ, Rubin MA, Chinnaiyan AM (2005) Recurrent fusion of TMPRSS2 and ETS transcription factor genes in prostate cancer. Science 310: 644–648 16254181 10.1126/science.1117679

[bib17] Tu JJ, Rohan S, Kao J, Kitabayashi N, Mathew S, Chen YT (2007) Gene fusions between TMPRSS2 and ETS family genes in prostate cancer: frequency and transcript variant analysis by RT-PCR and FISH on paraffin-embedded tissues. Mod Pathol 20: 921–928 17632455 10.1038/modpathol.3800903

[bib18] Valadi H, Ekstrom K, Bossios A, Sjostrand M, Lee JJ, Lotvall JO (2007) Exosome-mediated transfer of mRNAs and microRNAs is a novel mechanism of genetic exchange between cells. Nat Cell Biol 9: 654–659 17486113 10.1038/ncb1596

[bib19] van Gils MPMQ, Hessels D, van Hooij O, Jannink SA, Peelen WP, Hanssen SLJ, Witjes JA, Cornel EB, Karthaus HFM, Smits GAHJ, Dijkman GA, Mulders PFA, Schalken JA (2007) The time-resolved fluorescence-based PCA3 test on urinary sediments after digital rectal examination; a Dutch multicenter validation of the diagnostic performance. Clin Cancer Res 13: 939–943 17289888 10.1158/1078-0432.CCR-06-2679

